# Inhibition of MiR‐106b‐5p mediated by exosomes mitigates acute kidney injury by modulating transmissible endoplasmic reticulum stress and M1 macrophage polarization

**DOI:** 10.1111/jcmm.17848

**Published:** 2023-07-20

**Authors:** Xiang Li, Yanan Zhong, Rui Yue, Juan Xie, Yiyuan Zhang, Yongtao Lin, Hailun Li, Yong Xu, Donghui Zheng

**Affiliations:** ^1^ Department of Nephrology The Affiliated Huai'an Hospital of Xuzhou Medical University and Huai'an Second People's Hospital Huai'an China; ^2^ Department of Clinical Laboratory The Affiliated Huai'an Hospital of Xuzhou Medical University and Huai'an Second People's Hospital Huai'an China; ^3^ School of Nursing and Midwifery Jiangsu College of Nursing Huai'an China

**Keywords:** acute kidney injury, endoplasmic reticulum stress, exosomes, ischemia–reperfusion, macrophages

## Abstract

Acute kidney injury (AKI), mainly caused by Ischemia/reperfusion injury (IRI), is a common and severe life‐threatening disease with high mortality. Accumulating evidence suggested a direct relationship between endoplasmic reticulum (ER) stress response and AKI progression. However, the role of the transmissible ER stress response, a new modulator of cell‐to‐cell communication, in influencing intercellular communication between renal tubular epithelial cells (TECs) and macrophages in the AKI microenvironment remains to be determined. To address this issue, we first demonstrate that TECs undergoing ER stress are able to transmit ER stress to macrophages via exosomes, promoting macrophage polarization towards the pro‐inflammatory M1 phenotype in vitro and in vivo. Besides, the miR‐106b‐5p/ATL3 signalling axis plays a pivotal role in the transmission of ER stress in the intercellular crosstalk between TECs and macrophages. We observed an apparent increase in the expression of miR‐106b‐5p in ER‐stressed TECs. Furthermore, we confirmed that ALT3 is a potential target protein of miR‐106b‐5p. Notably, the inhibition of miR‐106b‐5p expression in macrophages not only restores ATL3 protein level but also decreases transmissible ER stress and hinders M1 polarization, thus alleviating AKI progression. Additionally, our results suggest that the level of exosomal miR‐106b‐5p in urine is closely correlated with the severity of AKI patients. Taken together, our study sheds new light on the crucial role of transmissible ER stress in the treatment of AKI through the regulation of the miR‐106b‐5p/ATL3 axis, offering new ideas for treating AKI.

## 1 INTRODUCTION

Ischemia/reperfusion injury (IRI) is one of the most common causes of acute kidney injury (AKI), primarily arising from factors such as transplantation, trauma, surgery, haemorrhage and shock.^1^, ^2^ It was established that in the injured renal microenvironment, renal tubular epithelial cells could regulate the destructive activity of macrophages by altering macrophage activation, proliferation, polarization and migration.^3^, ^4^ Interestingly, macrophages can also affect the viability of renal tubular epithelial cells by modulating the inflammatory response, remodelling tissue structure and cleaning cellular debris.^5^, ^6^ Nevertheless, the potential crosstalk mechanisms between damaged renal tubular epithelial cells and macrophages in the development of AKI remain not fully elucidated.

Endoplasmic reticulum (ER) stress, mainly caused by hypoxia, nutrient deprivation or nephrotoxin exposure, plays a crucial role in the progression of AKI.^7^ Exposed to ER stress, the unfolded protein response (UPR), an adaptive signalling pathway, is initiated and then engaged in transcriptional, post‐transcriptional and translational programmes, aiming at decreasing the number of nascent proteins translocated in the ER and elevating the ER protein folding capacity for maintaining ER homeostasis.^7^ In addition, the UPR also is in control of non‐cell autonomous responses, including inflammation, angiogenesis and tissue remodelling, which are essential mediators of human nephrogenic disease progression.^8^, ^9^ Fan et al.^10^ identified that the ER stress markers were closely correlated with the severity of AKI patients. Peyrou and Cribb revealed that pretreatment of renal tubular epithelial cells using ER stress inducers could significantly ameliorate AKI progression.^7^ Besides, Zhang et al.^11^ suggested that inhibition of the ER stress during ischemia–reperfusion induced AKI via erythropoietin‐derived peptide may protect renal tubular epithelial cells from injuries. Despite the compelling evidence presented by these studies, it is still challenging and intractable to look into the role of ER stress in the progression of AKI.

Recent studies demonstrated that ER‐stressed tumour cells could transmit similar ER stress and functional changes to recipient cells via certain ‘soluble factors’ released from donor cells.^12^ This concept was defined as transmissible ER stress (TERS).^13^ Exosomes, lipid membrane delimited vesicles secreted by the majority of cells, have emerged as new mediators involved in intercellular communication by transferring biologically active cargos between distinct cell types, like protein and nucleic acid contents.^5^ Accumulating evidence suggested that exosomes could be a contributory factor in transmitting ER stress, resulting in a series of relevant alterations of cellular functions. As it circumvents the likelihood of immune rejection and vascular occlusion,^14^ this strategy via exosomes to manipulate TERS may become a promising therapeutic approach for treating AKI. However, it is hitherto unclear whether TERS exists between renal tubular epithelial cells and macrophages in I/R‐AKI.

In the present study, we first demonstrated that H/R‐induced ER stress in renal tubular epithelial cells could be transmitted to macrophages via exosomes and simultaneously promote M1‐type polarization of macrophages. In addition, the genomic findings revealed that the miR‐106b‐5p/ATL3 signalling axis plays a pivotal role in the transmissible ER stress between tubular epithelial cells and macrophages. Besides, we confirmed that ALT3 is a potential target protein of miR‐106b‐5p. By inhibiting the expression of exosomal miR‐106b‐5p, the transmissible ER stress and the M1‐type polarization of macrophages could be dramatically inhibited, thereby ameliorating the injury and progression of renal tissue in mice models of I/R‐AKI. Taken together, our study presents novel insights into the role of transmissible ER stress in intercellular communication in the regulation of the progression of I/R‐AKI.

## 2 MATERIALS AND METHODS

### 2.1 Cell culture and the establishment of cell hypoxia/reoxygenation (H/R) model

The mouse renal tubular epithelial cell line (mRTEC), human renal tubular epithelial cell line (HK‐2), mouse mononuclear macrophage leukaemia cell line (RAW264.7), and human monocytic leukaemia cell line (THP‐1) were all purchased from Shanghai WheLab Company. All cell lines were cultured in DMEM (Invitrogen, USA) supplemented with 10% FBS (Gibco, United States) and 1% penicillin–streptomycin (Gibco, GrandIsland, NY) at 37°C, 5% CO_2_. THP‐1 differentiation into M0 macrophages was induced using phorbol ester.

Cellular H/R damage model was established according to the previously described methods.^47^ Briefly, the hypoxic injury was induced by culturing mRTEC or HK‐2 cells in nutrient‐free (glucose‐free, serum‐free) in hypoxic conditions (1% O_2_, 5% CO_2_, 94% N_2_) for 6 h. The media was then replaced with fresh nutrient medium supplemented with 10% FBS and the cells were cultured in normoxic conditions (5% CO_2_, 37°C) for 12 or 24 h. Control cells were cultured in normoxic conditions.

### 2.2 ER stress‐conditioned medium and co‐culture system

To produce ER stress‐conditioned medium (c.m.), TECs were first confirmed to be in ER stress using ER stress marker protein detection. Their cell culture media was then collected and centrifuged at 3000 *g* for 1 min and filtered using a 0.22 μm filter to remove cell debris and obtain the ER‐stressed TECs conditioned media. To simulate the paracrine effects of ER‐stressed TECs on macrophages, we cultured the macrophages in the TEC‐conditioned media. Briefly, the macrophages were rinsed thrice using PBS and then cultured in the ER‐stressed TEC‐conditioned media for 24 h at 37°C, 5% CO_2_.

### 2.3 Exosome isolation, identification and uptake

Exosomes were isolated using differential centrifugation and filtration.^48^ Briefly, culture media from renal tubular epithelial cells were centrifuged at 300 ***g*** for 20 min, 2500 ***g*** for 30 min, and 10,000 ***g*** for 30 min. They were then filtered through a 0.22 μm pore filter (Millipore, USA) and diluted with 120,000 ***g*** (Beckman Coulter Optima XPN‐100). Exosomes were then rinsed with PBS, filtered through a 0.22 μm filter, centrifuged at 120,000 ***g*** (Beckman Coulter Optima XPN‐100) for 70 min, resuspended in 200 μL PBS and stored at −80°C.

The morphological characteristics of the exosomes were assessed on a transmission electron microscope (JEM‐2100F, Japan) and their sizes were determined via nanoparticle tracking analysis (NTA) on a Zeta View PMX120 (Particle Metrix; Meerbusch, Germany) as previously described.^48^ Exosome isolation was confirmed via western blot analysis of the exosomal markers CD63 (ab216130, Abcam), TSG101 (ab125011, Abcam), and Alix (ab275377, Abcam).

For exosome uptake experiments, TECs‐derived exosomes were prepared using a Red Fluorescent Cell Linker Kit (Sigma Aldrich) according to manufacturer guidelines. Briefly, 4 μL of PKH26 dye (Sigma Aldrich) was added to the exosome suspension and incubated for 30 min to obtain red fluorescent‐labelled exosomes at room temperature. The exosomes were then washed with PBS to remove the unbound dye. The fluorescently labelled exosomes were ultra‐centrifuged at 120,000 ***g*** (Beckman Coulter optima xpn‐100) for 120 min, and thereafter, exosomes were incubated with macrophages for 24 h. Next, the cells were fixed with 4% paraformaldehyde (p0099, beyotime) for 20 min and then incubated with phalloidin (49409, sigma Aldrich) for 40 min and DAPI (ab285390, Abcam) for 30 min. Before adding the exosomes, the cells were washed thrice (2 min per wash) using PBS. Finally, exosomal uptake was examined under a confocal microscope (fv1000, Olympus, Japan).

### 2.4 Animals and treatment

Male C57BL/6 mice were purchased from Charles River. All animal experiments were approved by the ethics committee of Xuzhou Medical University. Treatment of mice with exosomes was done as described previously.^40^ Briefly, mice were anaesthetised via intraperitoneal injection of 0.1% pentobarbital sodium (50 mg/kg). Next, the kidneys were exposed and injected at five randomly selected sites with exosomes (50 μg/mL) from TECs transfected with miR‐106b‐5p inhibitor or NC‐inhibitor. To establish a model of renal ischemia/reperfusion (I/R) injury, bilateral I/R injury was induced by clamping the renal pedicle for 30 min, and thereafter the mice were euthanized after 24 h.

### 2.5 Clinical study and patients

All study participants gave written informed consent for urine donation. Ethical approval for the study was granted by the Affiliated Huai'an Hospital of Xuzhou Medical University. Cardiac surgery was done in accordance with standard clinical practice. Based on the KDIGO criteria,^41^ cardiac surgery‐associated acute kidney injury (CSA‐AKI) is defined as an increase in blood SCr levels to ≥26.5 μM within 48 h after surgery. Thirty‐six CSA‐AKI patients were classified into stage I (*n* = 16), stage II (*n* = 11) and stage III (*n* = 9). Patients with congestive heart failure, chronic obstructive pulmonary disease, infectious diseases, end‐stage renal disease, diabetes requiring insulin, preoperative proteinuria, emergency surgery, repeat cardiac surgery due to bleeding and patients diagnosed with AKI after 48 h were excluded from the study.

### 2.6 Statistical analysis

Statistical analysis was done on SPSS 22.0. Measurement data with continuous variables and normal distribution were expressed as mean ± SD. Data with non‐normal distribution were expressed as median or interquartile range (IQR). Differences between the two groups were compared using Student's *t* test or Mann–Whitney *U*‐test. One‐way anova with Bonferroni correction was used to compare differences between more than two groups. Correlation analysis was done using Pearson correlation coefficient. The diagnostic efficacy of the observation indexes was evaluated using receiver operating characteristic (ROC) curve analysis. *P* < 0.05 was considered statistically significant.

## 3 RESULTS

### 3.1 The ER stress could be transmitted from TECs to macrophages in vitro

**FIGURE 1:**
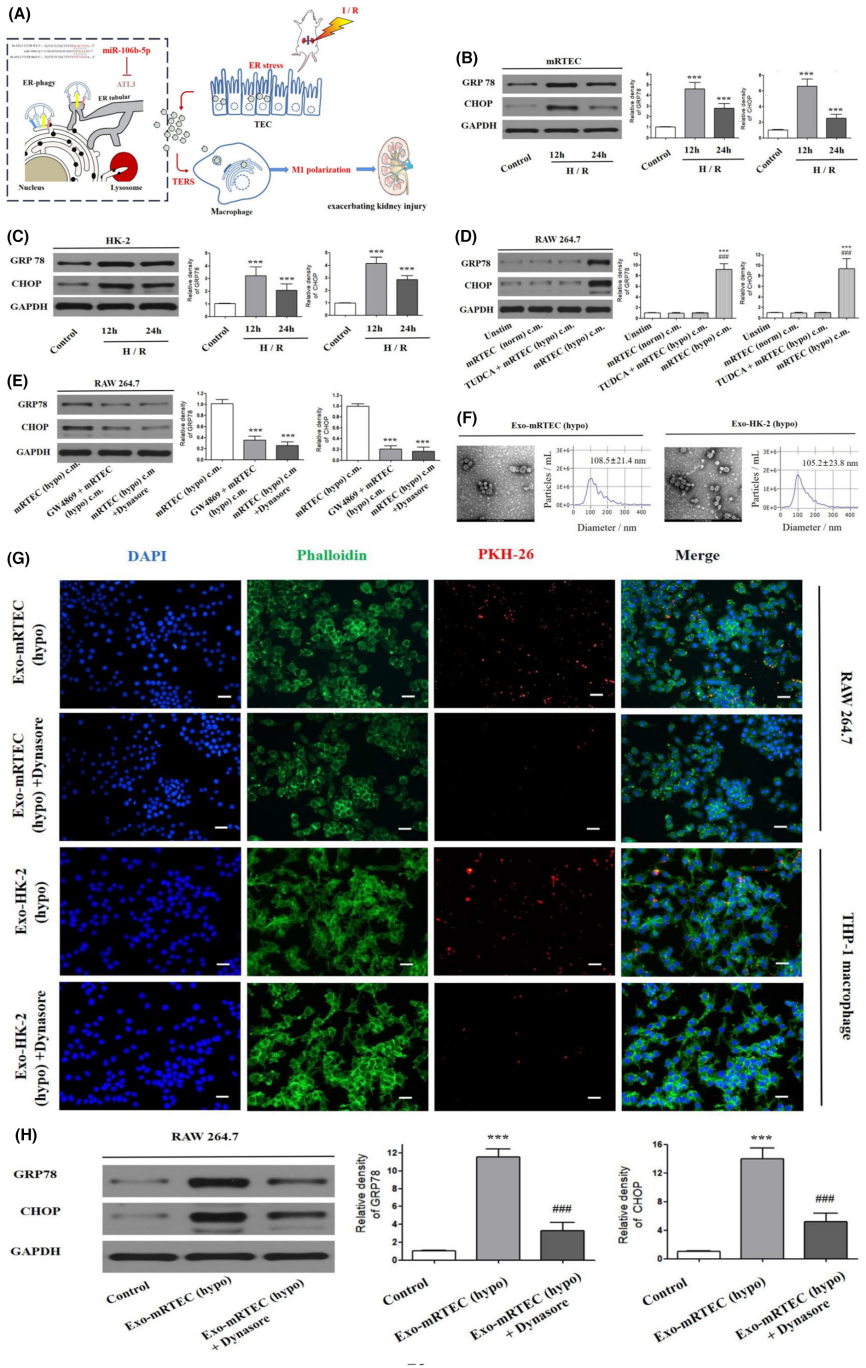
The ER stress could be transmitted from TECs to macrophages via exosomes in vitro. (A) Schematic illustration of the underlying mechanism of transmissible ER stress from TECs to macrophage that attenuates I/R‐induced AKI development. The protein levels of the ER stress markers, GRP78 and CHOP in the mRTEC (B) and HK‐2 cells (C) subjected to H/R using Western blot. (D) Western blot quantifications of protein levels of the ER stress markers, GRP78 and CHOP, in RAW264.7 macrophages cultured in the different conditioned media (c.m.) from ER‐stressed mRTECs [mRTEC (hypo)], mRTECs without ER stress [mRTEC (norm)], and ER‐stressed mRTECs treated with TUDCA [TUDCA+mRTEC (hypo)] for 12 h. Unstim macrophages were cultured in normal media for 12 h. (E) Western blot analysis of GRP78 and CHOP expression levels in RAW264.7 cultured in conditioned media from mRETC pretreated with GW4869 (10 μM) or Dynasore (50 μM) for 12 h. *** indicates *P* < 0.001 compared with the mRTEC (hypo) c.m. group. (F) TEM imaging and NTA analysis of exosomes isolated from conditioned media from ER‐stressed mRTEC or HK‐2. Scale bar: 200 nm. (G) Fluorescence microscopy images of PKH26‐labelled exosomes (red signals) in macrophages pretreated with or without dynasore for 12 h. Phalloidin‐labelled F‐Actin cytoskeleton (green) and DAPI‐labelled nuclei (blue). Scale bar: 20 μm. (H) Western blot analysis of GRP78 and CHOP levels in recipient RAW264.7 macrophages incubated with mRTECs‐derived exosomes (30 ng/mL) for 12 h.

To determine whether transmissible ER stress exists between TECs and macrophages, we first evaluated the status of H/R‐induced ER stress in the mouse‐derived mRTEC cells [mRTEC (hypo)] and human‐derived HK‐2 cells [HK‐2 (hypo)]. Figure 1A displays a schematic illustration of the role of transmissible ER stress in impacting intercellular communication between TECs and macrophages, thereby exacerbating I/R‐induced AKI development. Western blot results demonstrated that the protein expression levels of the ER stress markers, glucose‐regulated protein 78 (GRP78) and CCAAT/enhancer‐binding protein homologous protein (CHOP), were apparently elevated in mRTEC (hypo) cells and HK‐2 (hypo) cells after H/R induction for 12 h and 24 h, in which the effect was more pronounced after 12 h of treatment (Figure 1B,C).

Afterward, to verify if the ER stress could be transmitted from TECs to macrophages, we cultured RAW264.7 macrophages in the conditioned media (c.m.) from ER‐stressed mRTEC cells [mRTEC (hypo) c.m.]. Figure 1D suggested that when compared with unstimulated RAW264.7 cells (Unstim group), the expression level of GRP78 and CHOP proteins were significantly elevated in the mRTEC (hypo) c.m. group, verifying the activation of ER stress (*P* < 0.001). However, the treatment of mRTEC (norm) c.m did not significantly alter the expression levels of GRP78 and CHOP proteins in RAW264.7 cells (*P* > 0.05). To make sure that ER stress in the recipient macrophages was only induced by ER‐stressed TECs and not by other factors, we pretreated donor mRTECs with tauroursodeoxycholic acid (TUDCA, 25 μM) to inhibit ER stress prior to H/R (Figure 1D). Notably, the ER stress levels were significantly lower in macrophages after co‐treatment with TUDCA + mRTEC (hypo) c.m. than that in the mRTEC (hypo) c.m. group (*P* < 0.05). Similar observations were also verified in THP‐1 macrophage cells treated with HK‐2 (hypo) c.m. (*P* < 0.05, Figure S1). Taken together, these results above indicate that ER stress could be transmitted from TECs to macrophages.

### 3.2 Exosomes act as vehicles to transmit ER stress from TECs to macrophages

To assess whether exosomes can act as vehicles to transmit ER stress from TECs to macrophages, we evaluated it by inhibiting exosome production in donor cells or suppressing endocytosis in recipient cells. First, the exosome production was inhibited by pretreating mRTEC cells with the ceramide inhibitor, GW4869 + mRTEC (hypo) c.m., before H/R induction. Figure 1E showed that when compared with mRTEC (hypo) c.m. group, the protein expression levels of GRP78 and CHOP were significantly decreased in RAW264.7 cells treated with GW4869 + mRTEC (hypo) c.m. (*P* < 0.05). Next, to block endocytosis, the recipient RAW264.7 macrophages were pretreated with dynasore, an inhibitor of clathrin‐ and caveolin‐dependent endocytosis, before being treated with TECs c.m. Figure 1F,G showed that the number of produced exosomes in RAW264.7, as well as the protein levels of GRP78 and CHOP, was significantly increased upon co‐treatment of mRTEC (hypo) c.m. and dynasore (*P* < 0.05). Similar findings were also observed in THP‐1 macrophages after co‐treatment with HK‐2 (hypo) c.m. + Dynasore (both *P* < 0.05, Figures S2 and 1G).

Additionally, we isolated and purified exosomes from the supernatants of TECs. Transmission electron microscopy (TEM) images shown in Figure 1F indicated that mRTEC‐derived exosomes [Exo‐mRTEC (hypo)] and HK‐2‐derived exosomes [Exo‐HK‐2 (hypo)] had typical vesicular structures with the average diameters of 108.5 ± 21.4 nm and 105.2 ± 23.8 nm, respectively. Western blot results shown in Figure S3 revealed the expression of typical exosomal markers CD63, TSG101, and Alix. As expected, the Exo‐mRTEC (hypo) and Exo‐HK‐2 (hypo) were effectively internalized by ‘parent’ macrophages (Figure 1G), thereby potentially triggering ER stress in macrophages, as evidenced by significantly upregulated levels of GRP78 and CHOP (all *P* < 0.05, Figures 1H and S4). These results collectively indicate that ER‐stressed TECs are able to transmit ER stress to macrophages by exosomes.

### 3.3 Transmissible ER stress transferred by EVs promotes M1 polarization of macrophage

**FIGURE 2:**
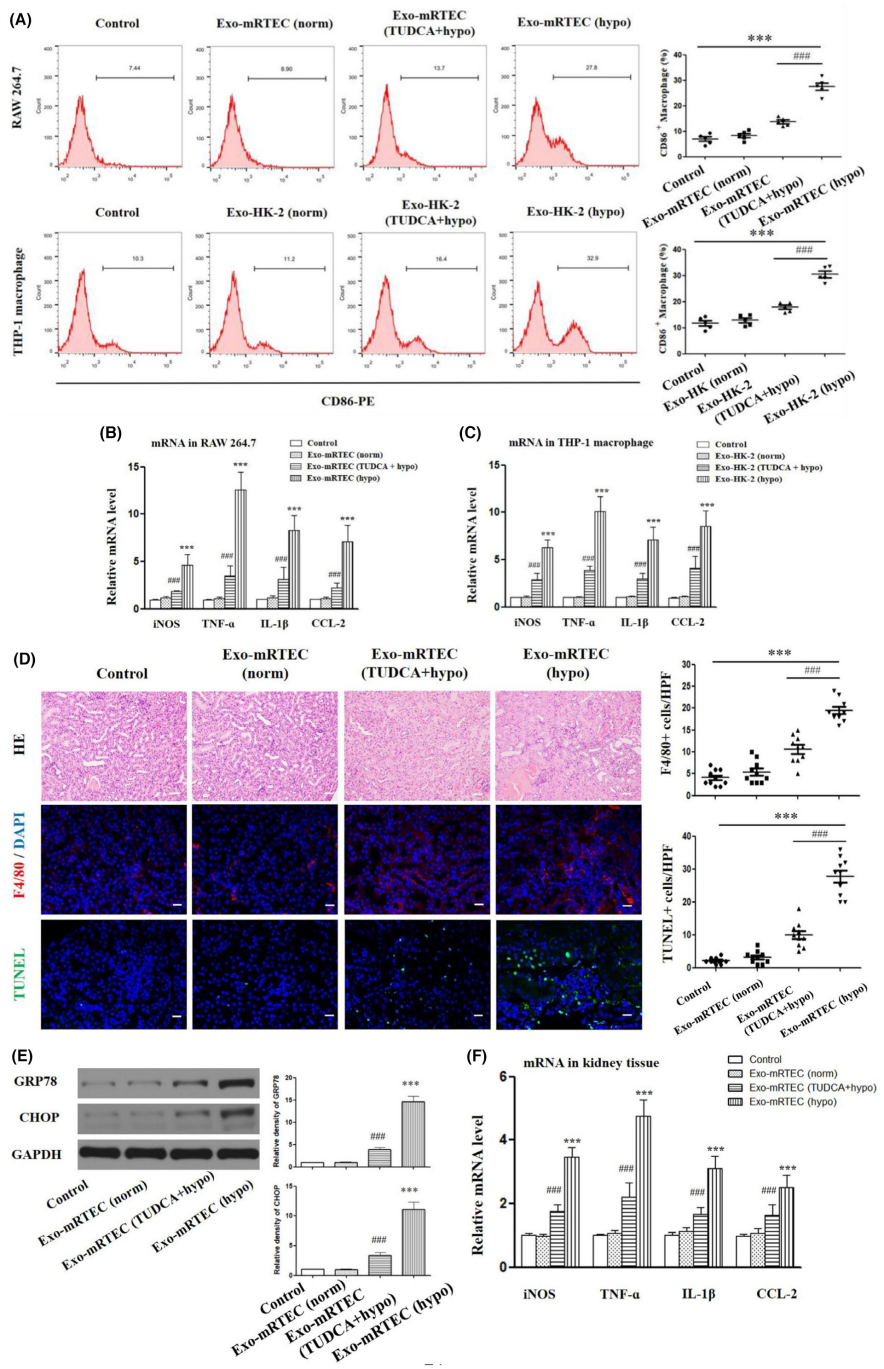
Transmissible ER stress transferred by exosomes promotes M1 polarization of macrophage. (A–C) Macrophages were incubated with exosomes from normoxic or hypoxic TECs (mRTEC/HK‐2) with or without 12 h of TUDCA treatment. Macrophages treated with an equal volume of PBS were the control. (A) Determination of the proportion of CD86‐positive macrophages using flow cytometry. (B, C) The mRNA levels of iNOS, TNF‐α, IL‐1β, and CCL‐2 in RAW264.7 and THP‐1 macrophages via RT‐PCR. (D‐F) TECs‐derived exosomes (50 μg/mL) or the same volume of PBS (200 μL) were injected into the kidneys of BALB/c mice. (D) H&E staining, immunohistochemical staining of F4/80 expression in macrophages, TUNEL apoptosis staining of kidney tissue injected with TECs‐derived exosomes (50 μg/mL) or PBS (200 μL). The number of F4/80‐ and TUNEL‐positive cells were quantified. Scale bar: 40 μm. (E) Western blot analysis of levels of GRP78 and CHOP in renal cortex. (F) RT‐PCR analysis of expression levels of iNOS, TNF‐α, IL‐1β, and CCL‐2 in renal cortex. *** indicates *P* < 0.001, compared with the Control. ^###^ indicates *P* < 0.001 compared with the Exo‐TEC (hypo) treatment group.

Next, we investigated the impact of ER‐stressed TECs‐derived exosomes on macrophage phenotype variations. In vitro experiments revealed that when compared with the control group (macrophages without exosomes treatment), the rate of CD86 positive cells, the mRNA expression levels of iNOS (a popular M1 macrophage marker) and pro‐inflammatory factors TNF‐α, IL‐1β, and CCL‐2 were significantly higher in RAW264.7 macrophages treated with Exo‐mRTEC (hypo) and THP‐1 macrophages treated with Exo‐HK‐2 (hypo) (Figure 2A–C, all *P* < 0.05). Nonetheless, there were no apparent variations in these indicators in the Exo‐mRETC (norm) group and Exo‐HK‐2 (norm) group (*P* > 0.05). The in vivo immunofluorescence observations shown in Figure 2D revealed a significant increase in F4/80 positive macrophage infiltration in the kidneys treated with local injection with Exo‐mRTEC (hypo) for 24 h (*P* < 0.05). Consistent with these observations, the lesion extent of renal tissue and the apoptosis level were significantly increased in the Exo‐mRTEC (hypo) treatment group (Figure 2D).

To determine whether the phenotype changes of macrophages were associated with the ER stress in TECs via exosomes transfer, we pretreated mRTECs or HK‐2 cells with TUDCA, an ER stress inhibitor, and then isolated their exosomes, Exo‐mRTEC (TUDCA+hypo) and Exo‐HK‐2 (TUDCA+hypo) from the conditioned media. Figure 2A–C showed that the number of CD86 positive cells and the mRNA levels of iNOS, TNF‐α, IL‐1β and CCL‐2 were significantly decreased upon exposure of Exo‐mRTEC (TUDCA+hypo) and Exo‐HK‐2 (TUDCA+hypo) exosomes to RAW274.6 and THP‐1 macrophages (*P* < 0.05). Moreover, it was revealed that the renal tissue lesions were significantly ameliorated in the Exo‐mRTEC (TUDCA+hypo) group compared with the Exo‐mRTEC (hypo) group, accompanied by significantly decreased infiltration by F4/80‐positive macrophages, cytokine mRNA levels, apoptosis rates, and protein levels of GRP78 and CHOP (Figure 2D–F). These findings suggested that the exosomes derived from TECs undergoing ER stress may potentially transmit ER stress to macrophages and simultaneously induce their M1‐type polarization.

### 3.4 Significantly elevated MiR‐106b‐5p in exosomes derived from ER‐stressed TECs may be involved in transmissible ER stress

**FIGURE 3:**
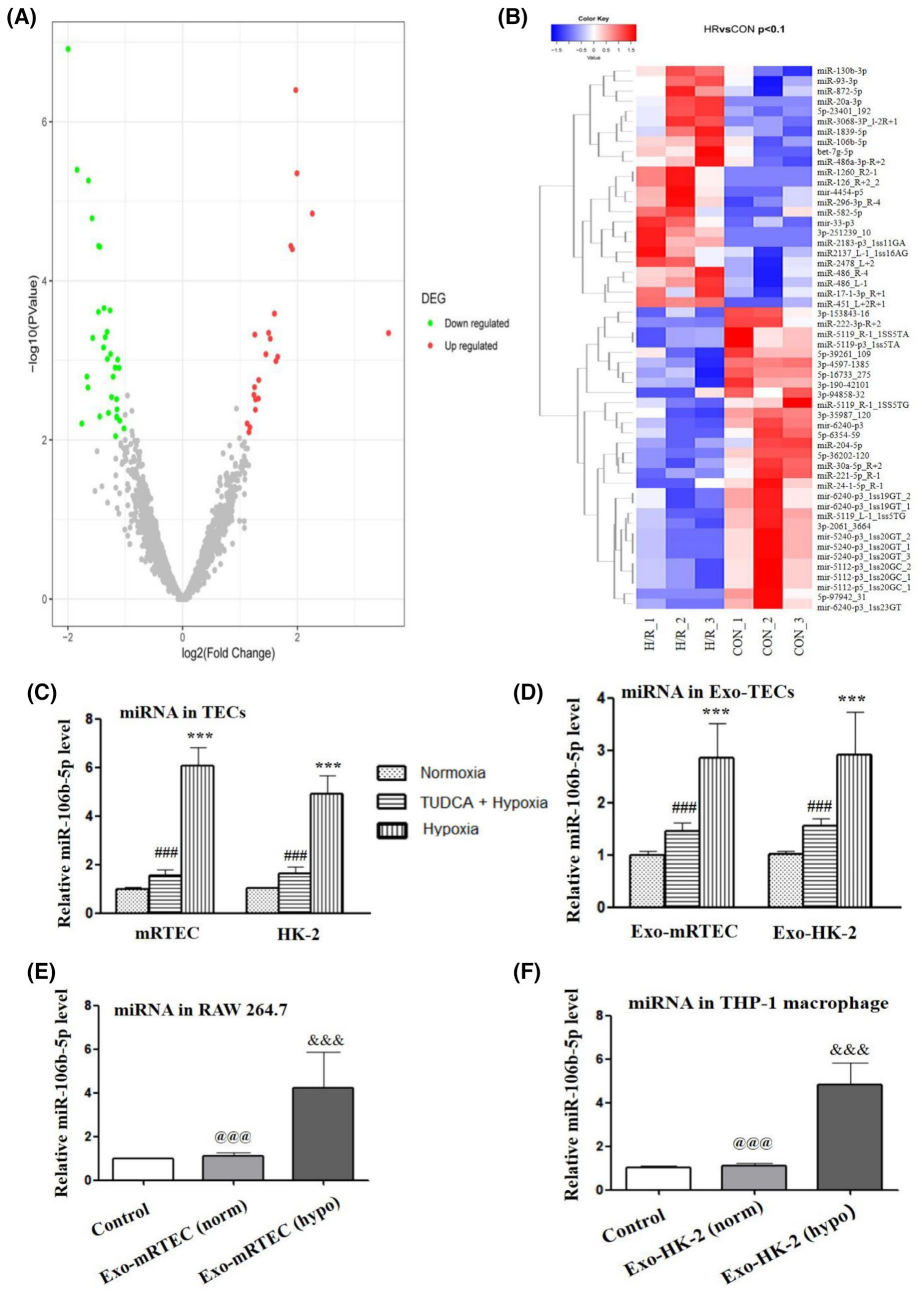
Significantly elevated MiR‐106b‐5p level in exosomes derived from ER‐stressed TECs. (A) Volcano map of miRNA‐seq data identifying differentially expressed miRNAs in exosomes derived from TECs (control group versus H/R group). Red dots, significantly upregulated miRNAs. Blue dots, significantly downregulated miRNAs. Grey dots, non‐differentially expressed miRNAs. (B) Visualization of clustering and heatmap showing the differentially expressed miRNAs. Red signals and blue signals stand for upregulated and downregulated expression, respectively. RT‐PCR analysis of selected miR‐106b‐5p expression levels in the TECs (C), TECs‐derived exosomes (D), exosome‐treated RAW264.7 macrophages (E), and exosome‐treated THP‐1 macrophages (F). Nomoxia indicates cells are cultured in normoxic conditions. Hypoxia indicates cells undergo H/R treatment. TUDCA+hypoxia indicates cells receiving TUDCA treatment undergo H/R. *** indicates *P* < 0.001 compared with nomoxia group. ^###^ indicates *P* < 0.001 compared with hypoxia group, ^&&&^ indicates *P* < 0.001 compared with control group, ^@@@^ indicates *P* < 0.001 compared with Exo‐TEC (hypo) group.

To elucidate the underlying mechanism involving transmissible ER stress between TECs‐derived exosomes and macrophages, we performed miRNA sequencing analysis for exosomes derived from TECs in or out of ER stress status. As shown in Figure 3A, it was revealed that when compared with the Exo‐mRTEC (norm) control group, Exo‐mRTEC (hypo) group exhibited more than twofold differential expression in the 52 miRNAs, in which 22 miRNAs were upregulated, and 30 miRNAs were downregulated (*P* < 0.05; Log2Fc >1.0). By clustering analysis, we noted that the expression levels of miR‐130b‐3p, miR‐93‐3p, miR‐872‐5p, miR‐20a‐3p, miR‐106b‐5p, and miR‐486a‐3p were significantly elevated in the Exo‐mRTEC (hypo) group when compared to the control group, as indicated in Figure 3B. Meanwhile, we performed qRT‐PCR experiments and found that the expression levels of six differentially upregulated microRNAs in exosomes derived from mRETCs subjected to hypoxia treatment were significantly upregulated (Figure S9), with miR‐106b‐5p being the most significantly altered after hypoxia treatment. On the basis of these evidence, we reason that miR‐106b‐5p is the most likely to be involved in transmissible ER stress. Therefore, miR‐106b‐5p was selected in our study for further investigations.

Moreover, we evaluated the miR‐106b‐5p expression levels in the TECs and TECs‐derived exosomes by RT‐PCR. It was noted that when compared with the control group, miR‐106b‐5p expression had a 6.07 ± 1.05 fold increase in the hypoxic mRTEC cells [mRTEC (hypo)] and a 2.84 ± 1.68 fold increase in the hypoxic mRTEC‐derived exosomes [Exo‐mRTEC (hypo)] (Figure 3C,D). Similarly, the miR‐106b‐5p expression had a 4.83 ± 1.78 fold increase in the hypoxic HK‐2 cells [HK‐2 (hypo)] and a 2.86 ± 1.55 fold increase in hypoxic HK‐2‐derived exosomes [Exo‐HK‐2(hypo)] (*P* < 0.05). However, after ER stress inhibition in mRTEC and HK‐2 cells using TUDCA, the expression level of miR‐106b‐5p was significantly decreased in both TECs and TECs‐derived exosomes. Next, the analysis of the expression of miR‐106b‐5p in macrophages had a 4.30 ± 1.36 fold and 4.67 ± 1.59 fold increase in RAW 264.7 and THP‐1 macrophages receiving Exo‐mRTEC (hypo) or Exo‐HK‐2 (hypo) treatment, respectively (*P* < 0.05, Figure 3E,F). In addition, we also examined whether chemical activator‐induced ER stress (tunicamycin) could be transmitted between mRTEC and RAW264.7 cells (Figure S10). It was found that tunicamycin (Tm)‐induced ER stress could not be effectively transmitted from mRTEC to RAW264.7, as evidenced by not increased expression levels of GRP78 and CHOP. These results suggest that after ER stress occurred in TECs, the miR‐106b‐5p expression level was dramatically increased and subsequently can be effectively transferred to macrophages via exosomes derived from ER‐stressed TECs.

### 3.5 ATL3 is a potential target of miR‐106b‐5p and potentially participates in ER stress and M1 polarization of macrophages

**FIGURE 4:**
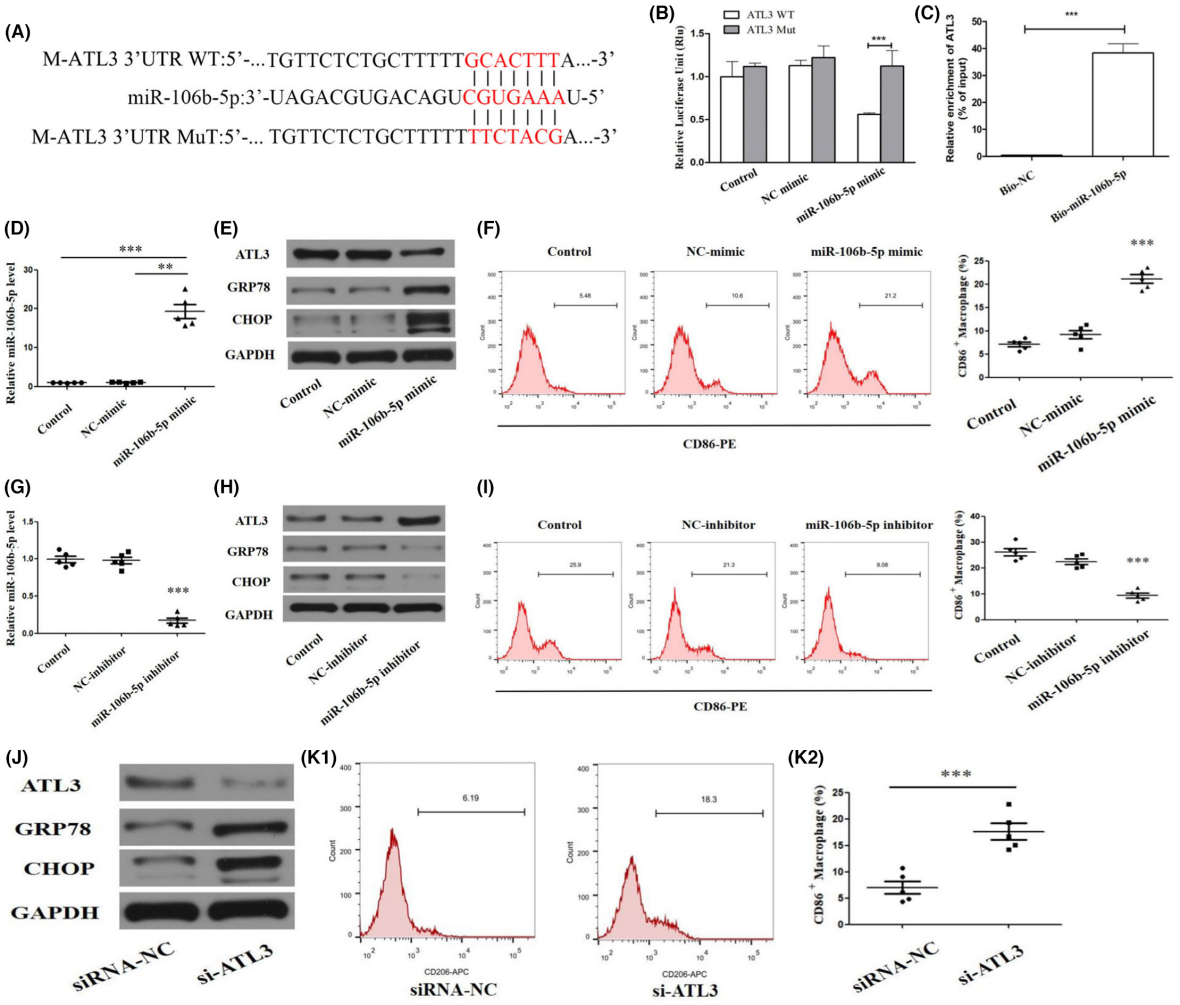
ATL3 is a potential target of miR‐106b‐5p, and potentially participates in ER stress and M1 polarization of macrophages. (A) Predicted binding site between miR‐106b‐5p and the 3′‐UTR ends of ATL3. (B) Potential binding between miR‐106b‐5p and the ATL3 3′‐UTR was demonstrated by the luciferase reporter assay. (C) The interaction between ATL3 and miR‐106b‐5p was evaluated by RNA pull‐down analysis. (D–F) RAW264.7 macrophages were incubated with the exosomes from mRTECs transfected with miR‐106b‐5p mimic or NC‐mimic for 12 h. Control exosomes were isolated from mRTECs with plasmid‐free transfection. (D) qRT‐PCR analysis for miR‐106b‐5p expression level in RAW264.7 macrophages. (E) Western blot analysis of the expression of ATL3, GRP78, and CHOP in macrophages determined by western blot. (F) Flow cytometric detection of CD86‐positive macrophages after miR‐106b‐5p inhibitor or NC inhibitor treatment. (G–I) ER‐stressed RAW264.7 macrophages were incubated with the exosomes from mRTECs transfected with miR‐106b‐5p inhibitor or NC inhibitor for 12 h. (G) qRT‐PCR analysis of miR‐106b‐5p expression levels in RAW264.7 macrophages. (H) Protein levels of ATL3, GRP78, and CHOP in macrophages determined by western blot. (I) Flow cytometric detection of CD86‐positive macrophages after miR‐106b‐5p inhibitor or NC inhibitor treatment. (J‐K) RAW264.7 macrophages were transfected with siRNA‐ATL3 or siRNA‐NC. si‐ATL3: 5′‐GACUAGUUCUGUUCAGAUUUA‐3′, 5′‐AAUCUGAACAGAACUAGUCAU‐3′. (J) Western blot analysis of ATL3, GRP78, and CHOP levels in macrophages. (K1–K2) Flow cytometric detection and quantification of CD86‐positive macrophages.

To explore the mechanism on how the miR‐106b‐5p is involved in transmissible ER stress, we predicted the downstream target genes by bioinformatics analysis of miRDB (https://mirdb.org/), miRTarbase (https://mirtarbase.cuhk.edu.cn/) and miRPathDB (https://mpd.bioinf.uni‐sb.de/). As shown in Figure 4A, it demonstrated the atlastin 3 (ATL3) as a potential target for the modulation of miR‐106b‐5p. To validate the regulatory effect of miR‐106b‐5p on ATL3, we performed a luciferase reporter gene assay and noted that the miR‐106b‐5p mimic significantly inhibited and decreased ATL3‐WT fluorescence signals but not ATL3‐Mut (Figure 4B). The miRNA pull‐down analysis of the interaction between miR‐106b‐5p and ATL3 revealed the enrichment of the ATL3 probe by biotinylated miR‐106b‐5p (Figure 4C). After mRTEC cells were transfected with miR‐106b‐5p mimic via liposome transfection technique, it was found that the miR‐106b‐5p expression level in mRTEC‐derived exosomes was significantly increased (Figure 4D). Notably, compared with RAW264.7 macrophages treated with the NC‐mimic, the ATL3 protein levels were significantly decreased in macrophages received with miR‐106b‐5p mimic, which was due to obviously elevated levels of miR‐106b‐5p (Figures 4E and S5). Figures 4G,H and S6 showed that the treatment of miR‐106b‐5p inhibitor obviously suppressed miR‐106b‐5p expressions in TECs‐derived exosomes, whereas it significantly reversed and elevated the ATL3 expression levels in recipient RAW264.7 macrophages. Meanwhile, we examined the variations in ATL3 levels in recipient RAW264.7 macrophages after TECs‐derived exosomes treatment (Figure S11). In comparison with the mRTECs‐derived exosomes (norm) group, there is a significant decrease in the expression levels of both ALT3 proteins and mRNA in the recipient macrophages after mRTECs‐derived exosomes (hypo) treatment for 24 h.

Next, we explored the effect of exosomal miR‐106b‐5p on macrophage ER stress and M1 macrophage polarization. Figure 4E revealed that the protein levels of GRP78, CHOP, and the number of CD86‐positive cells were significantly increased in the recipient macrophages after the treatment with the miR‐106b‐5p mimic for 12 h. As expected, the ER stress and M1‐type polarization in macrophages after treatment with miR‐106b‐5p inhibition were apparently inhibited, as evidenced by reduced protein levels of GRP78, CHOP, and the number of CD86‐positive cells (Figure 4H,I). ATL3, a key player in ER autophagy (ER‐phagy), plays a crucial role in the maintenance of ER homeostasis.^15^ Notably, we discovered that ATL3 silencing in macrophages (si‐ATL3 group) could trigger noticeable ER stress and induce M1 macrophage polarization relative to the siRNA‐NC group (Figure 4J–K2). These findings indicate that miR‐106b‐5p may trigger the ER stress and M1 polarization of macrophages by inhibiting the ATL3 expression levels.

### 3.6 Inhibition of miR‐106b‐5p expression could mitigate kidney damage in I/R‐AKI mice

**FIGURE 5:**
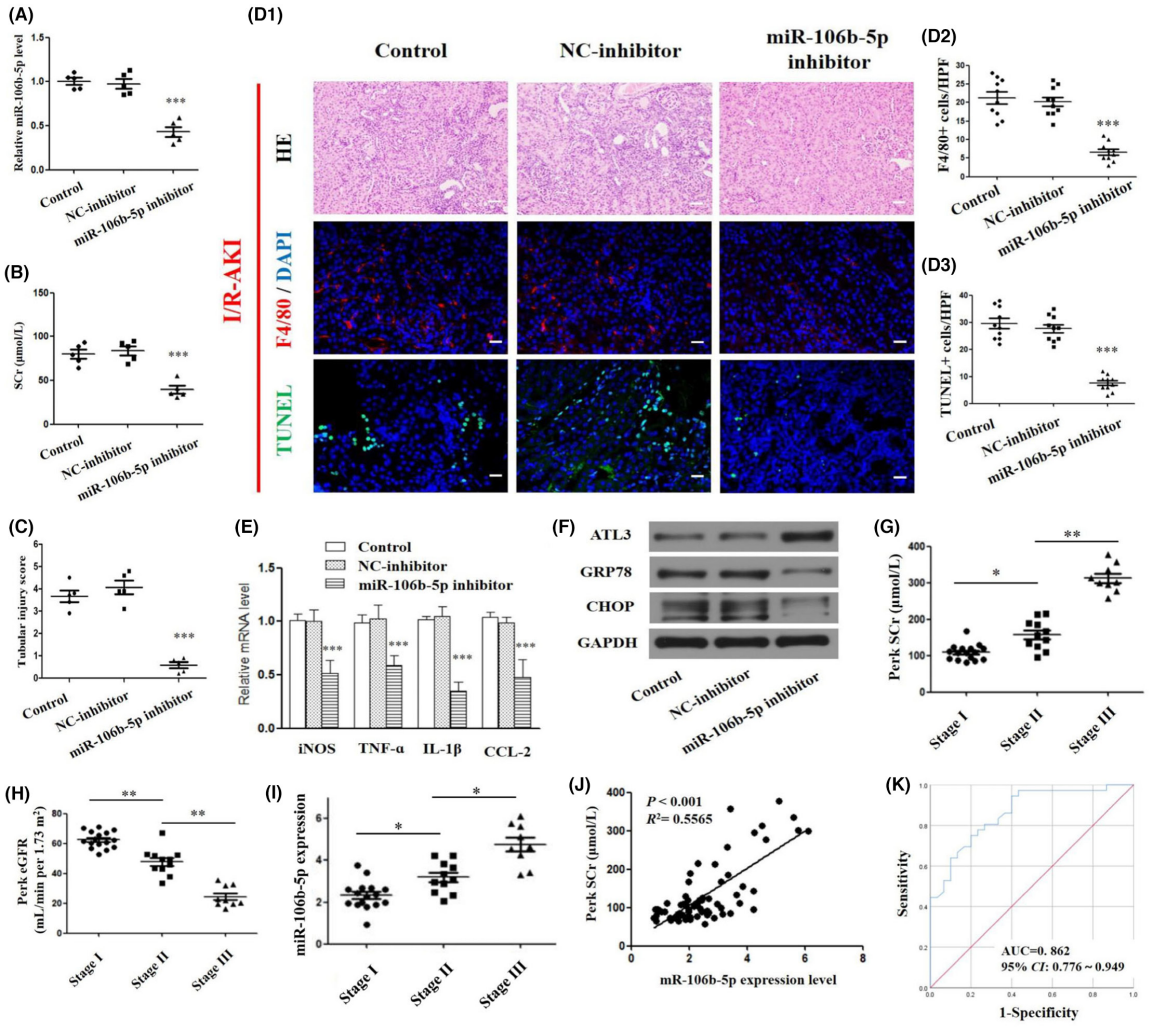
Inhibition of miR‐106b‐5p expression mitigates kidney damage in I/R‐AKI mice. The exosomes derived from mRTECs transfected with miR‐106b‐5p inhibitor or NC‐inhibitor were locally injected into the kidneys of I/R‐AKI mice. (A) The miR‐106b‐5p expression in kidney tissue determined by RT‐PCR. Detection of SCr (B), and renal tubular injury score (C) of kidneys after various treatments. (D1) H&E staining, F4/80 immunofluorescence staining, and TUNEL assay of kidney tissues after various treatments. (D2–D3) Quantification of the number of F4/80‐positive cells (right histogram) and TUNEL‐positive cells (right histogram). Scale bar: 40 μm. (E) RT‐PCR analysis of the mRNA levels of iNOS, TNF‐α, IL‐1β, and CCL‐2 in kidney tissues after various treatments. (F) Western blot analysis of the expression of ATL3, GRP78, and CHOP in kidney tissues after various treatments. Comparison of the levels of SCr (G), peak eGRR (H), and urinary miR‐106b‐5p (I) in patients with different CSA‐AKI stages. Analysis of the correlation between miR‐106b‐5p levels and peak Scr (J). ROC curve analysis (K) of the diagnostic value of miR‐106b‐5p levels within urinary exosomes in CSA‐AKI.

Given the above exciting evidence, we further validated the in vivo therapeutic efficacy of the miR‐106b‐5p for AKI. mRTECs were transfected with the miR‐106b‐5p inhibitor or NC‐inhibitor, and we then isolated their exosomes derived from mRETCs and locally injected them into the kidneys of mice. These mice were eventually subjected to I/R surgery and euthanized after 24 h. It was observed that the treatment of miR‐106b‐5p inhibitor, causing the inhibition of miR‐106b‐5p, not only led to significantly decreased levels of serum creatinine (SCr) but also improved renal injury scores and histopathological changes in I/R‐AKI mice (Figure 5A–D). Consistent with these results, it was noteworthy that treatment of miR‐106b‐5p inhibitor transported by exosomes also resulted in a significant decrease in the number of F4/80‐positive macrophages (Figure 5D1–D3) and the mRNA levels of M1 macrophage markers, iNOS, TNF‐α, IL‐1β and CCL‐2 (Figure 5E), suggesting the weaken activation of pro‐inflammatory M1‐type polarization of macrophages in the AKI models. Notably, there was a significant reduction in the protein levels of GRP78 and CHOP in kidney tissues of I/R‐AKI mice treated with miR‐106b‐5p inhibitor, while ATL3 protein expressions were in turn increased (Figures 5F and S7). These results demonstrate that miR‐106b‐5p inhibitor can be delivered to renal macrophages via TECs‐derived exosomes, causing the inhibition of miR‐106b‐5p, and functionally led to an apparent decrease of transmissible ER stress and M1 polarization of macrophages, thereby, ameliorating renal tissue injury and progression of I/R‐AKI. On the contrary, it was revealed that miR‐106b‐5p participates in transmissible ER stress between TECs and macrophages and promotes the M1 macrophage polarization by targeting and modulating ATL3 protein expressions.

### 3.7 Urinary exosomal miR‐106b‐5p may act as an applicable diagnostic biomarker for AKI patients

To explore the urinary exosomal miR‐106b‐5p as a diagnostic biomarker in AKI patients, we isolated exosomes from the urine of 66 patients who received cardiac surgery, including 36 cardiac surgery‐associated acute kidney injury (CSA‐AKI) patients. The clinical characteristics of the CSA‐AKI and non‐CSA‐AKI patients are exhibited in Table S2. The hospitalization time of the CSA‐AKI patients was significantly longer than that of the non‐CSA‐AKI patients (*P* > 0.05). Preoperative baseline comparison revealed no significant differences in the levels of SCr, eGFR, and haemoglobin (Hb) between the two groups (all *P* > 0.05). Compared with the non‐CSA‐AKI group, the postoperative SCr levels were elevated in the CSA‐AKI group, whereas eGRF levels were decreased (*P* < 0.01). Table S2 revealed that the relative expression level of miR‐106b‐5p in urinary exosomes from the CSA‐AKI group was 3.23 ± 1.06, which was significantly higher than that in the non‐CSA‐AKI group (1.77 ± 0.62, *P* < 0.001). The level of SCr significantly increased, whereas eGFR levels apparently fell in patients with stage I, II, and III CSA‐AKI (*P* < 0.05), reflecting significant differences in the degree of kidney injury across the stages. As given in Figure 5I, the levels of urinary exosomal mir‐106b‐5p levels gradually increased across stage I, II, and III CSA‐AKI patients (*P* < 0.05). Regarding Pearson correlation analysis (Figures 5J and S8), we found that miR‐106b‐5p levels in urinary exosomes were positively correlated with SCr levels with a correlation coefficient (*r*) of 0.746, while it was negatively correlated with eGFR levels with *r* = −0.751. ROC curve analysis shown in Figure 5K indicated that the expression level of urinary exosomal miR‐106b‐5p showed a favourable predictive value for CSA‐AKI diagnosis (AUC: 0.86, 95%, CI: 0.776–0.949, Youden index: 0.55, sensitivity: 75.0%, and specificity: 80.0%). All in all, urinary exosomal miR‐106b‐5p may potentially act as an applicable diagnostic biomarker for AKI patients.

## 4 DISCUSSION

The endoplasmic reticulum stress response and the UPR pathway are generally recognized as adaptive mechanisms that maintain endoplasmic reticulum homeostasis and support cell survival by elevating the protein folding capacity of the organelle. In the present study, we demonstrate for the first time that renal tubular epithelial cells can stimulate macrophage polarization towards the pro‐inflammatory M1 type via transmissible ER stress based on the I/R‐AKI model. In contrast, Bignon et al.^16^ reported that tunicamycin, thapsigargin or glucose starvation‐treated human lineage renal tubular epithelial cells failed to deliver ER stress to human monocyte‐derived macrophages. In our assays, it was further confirmed that tunicamycin induced a failure of ERS transmission from renal tubular cells to macrophages. This may be attributed to different induction strategies of ER stress. In addition to nutritional deficiency, renal tubular epithelial cells are susceptible to hypoxia.^17^ Moreover, shreds of evidence suggest that the transmissible ER stress is capable of afflicting phenotypic and functional alterations in neighbouring cells, depending largely on the pathophysiological context that triggers endoplasmic reticulum stress activation in donor cells, as well as on the specific cell type undergoing this process.^18^, ^19^, ^20^

Exosomes are crucial mediators to exert paracrine effects and can functionally regulate neighbouring or distant recipient cells.^5^, ^21^ Heusermann et al.^22^ indicated that the endoplasmic reticulum of recipient cells might be the principal site of exosome cargo release, as approximately 90% of exosomes have been shown to stagnate at or near the ER. Accordingly, we reason that exosomes may be involved in transmissible ER stress. More importantly, our study findings sufficiently substantiated our hypothesis since a reduction of exosome production in donor cells or impediment of endocytosis in recipient macrophages significantly decreases the sensitivity of macrophages to ER stress transmitted from renal tubular epithelial cells. Furthermore, we found that after the block of ER stress in renal tubular epithelial cells, their derived exosomes fail to induce ER stress in receptor macrophages. On the basis of these evidence, we are convinced that exosomes are involved in the transfer process of transmissible ER stress as a crucial mediator.

Macrophages are highly heterogeneous cell populations, and their phenotypes are demonstrated to be responsible for a variety of biological activities.^4^ Macrophages have functional activity status with distinct polarized conditions, usually pro‐inflammatory M1‐ or anti‐inflammatory M2‐phenotypes.^23^ It has been suggested that M1/M2 macrophage polarization status is an essential contributor that affects inflammatory response and fibrosis in determining the extent of renal parenchymal injury.^24^ Interestingly, we found that macrophages affected by transmissible ER stress could polarize towards the pro‐inflammatory M1 type, thereby eliciting the development of renal histological lesions. However, to the best of our knowledge, the exact components/signalling molecules responsible for transmissible ER stress have yet to be well understood so far.^25^ Several studies reported that transmissible ER stress could be activated and mediated by variations in extracellular concentrations of substances such as nucleic acids, proteins, and metabolites.^19^, ^26^ Our study found an apparent relationship between miR‐106b‐5p level and transmissible ER stress, in which there was a significantly increased miR‐106b‐5p level in the ER‐stressed renal tubular epithelial cells. Furthermore, we identified that exosomes could act as desirable vehicles for miR‐106b‐5p and transport it to macrophages. Based on this, it was convinced that the type and amount of exocytotic inclusions, especially miRNAs, are closely correlated with the ER stress status of donor cells. Consistent with our findings, inhibition of miR‐106b‐5p expression significantly ameliorated the progression in lipopolysaccharide (LPS)‐induced AKI.^27^ Besides, Antagonists targeting miR‐106b‐5p attenuate acute kidney injury by upregulating TCF4 to modulate renal function, apoptosis, and autophagy.^28^ A recent study suggested that miR‐106b‐5p expression levels were significantly increased in macrophages undergoing ER stress due to vitamin D deficiency,^29^ which is highly in accordance with our findings. Despite a certain amount of evidence presented by these studies, it was not explored how the regulatory relationship between miR‐106b‐5p and ER stress was precisely controlled.

ATL3, a member of the Dynamin superfamily, is a GTPase that influences motility and is a vital regulator of the structure and dynamics of the ER membrane.^30^ It has also been shown that ATL3 is implicated in the ER selective autophagy (ER‐phage), receptor‐mediated specific phagocytosis of damaged ER substructures and components.^31^, ^32^ To our best knowledge, proper protein folding is of critical importance for implementing normal protein function, and maintenance of the components and dimensions of ER membrane assists in protein homeostasis.^33^ ATL2 affects ER morphology primarily by promoting ER fusion, whereas changes in endoplasmic reticulum morphology are barely detectable after ATL3 depletion.^31^ Furthermore, ER stress and ER autophagy are two closely interlinked homeostatic processes; sustained ER stress activates the ER autophagy to clear the non‐functional ER fraction, including misfolded proteins; when the stress from the unfolded protein response subsides, ER autophagy could decrease the size of ER to its normal proportions.^34^, ^35^, ^36^ Previous studies revealed that ATL3 deletion significantly inhibits the degradation of tubular ER membrane proteins under starvation, and recombinant ATL3 has been shown to rescue the turnover of tubular ER proteins.^37^, ^38^ Additionally, ATL2/3 has been reported to directly promote the assembly of the ULK1 complex on the ER and to mediate the formation of autophagosomes.^37^ ATL2/3 knockout has been reported to significantly reduce the LC3 II/LC3 I ratio and inhibit autophagy.^31^ Therefore, aberrant ATL3 expression can disrupt the homeostatic balance of the ER, increasing cellular ERS sensitivity and severity, ultimately leading to the development of several diseases.

MiR‐106b‐5p, a member of the miR‐106b family, has been shown to be aberrantly expressed in numerous diseases and serves as a biomarker for early diagnosis and monitoring of disease progression, where there is generally persistent ER stress or dysfunctional ER stress mechanisms.^39^, ^40^, ^41^ For instance, miR‐106b‐5p underwent a 2.84‐fold increase in urinary extracellular vesicles (uEV) in patients with anti‐neutrophil cytoplasmic antibody (ANCA)‐associated vasculitis (AAV).^42^ In kidney tissue from patients with type 2 diabetes, miR‐106b‐5p was found to be reduced 3.42‐fold.^43^ In addition, it was similarly expressed abnormally in a number of ischaemic‐invasive diseases, which was a significant factor in triggering renal ER stress in our study. For patients with acute stroke, the level of miR‐106b‐5P was increased 3.63‐fold in MRI (−) patients and 23.90‐fold in MRI (+) patients^44^; the level of miR‐106b expression was significantly increased 4.6‐fold in patients with unstable angina (UA) who suffered an acute myocardial infarction.^45^ Moreover, the level of miR‐106b‐5p is an essential risk indicator for assessing cerebral ischemic events (CIE) in patients with asymptomatic carotid stenosis (CAS).^46^ In our study, we investigated the role of miR‐106b‐5p in the severity of CSA‐AKI patients. It was observed that the miR‐106b‐5p expression levels were significantly increased in patients with CSA‐AKI and correlated with the severity of CSA‐AKI compared to patients without CSA‐AKI. Nonetheless, our study still has the following limitations: (1) the sample size of the cohort was relatively small; (2) miR‐106b‐5p expression in renal tissue could not be measured since biopsies were not done on the patients. These factors limit the persuasiveness of the clinical findings to some extent.

## 5 CONCLUSION

In summary, we first demonstrated that H/R‐induced ER stress in renal tubular epithelial cells could be transmitted to macrophages via exosomes and simultaneously promote M1‐type polarization of macrophages in the progression of AKI. Besides, we identified that miR‐106b‐5p/ATL3 signalling axis plays a pivotal role in the transmissive ER stress in the intercellular communication between TECs and macrophages. Taken together, our study presents novel insights into the role of transmissive ER stress in intercellular communication in the regulation of I/R‐AKI progression, which offers a new possibility for AKI treatment against transmissible ER stress.

## AUTHOR CONTRIBUTIONS

**Xiang Li:** Conceptualization (equal); writing – original draft (equal). **Yanan Zhong:** Resources (equal); writing – original draft (equal). **Rui Yue:** Investigation (equal). **Juan Xie:** Resources (equal); software (equal). **Yiyuang Zhang:** Resources (equal); software (equal). **Yongtao Lin:** Investigation (equal); writing – review and editing (equal). **Hailun Li:** Formal analysis (equal); writing – review and editing (equal). **Yong Xu:** Conceptualization (equal); writing – review and editing (equal). **Donghui Zheng:** Conceptualization (equal); writing – review and editing (equal).

## FUNDING INFORMATION

This work was supported by National Natural Science Foundation of China (Grant Number 82170757), Science and Technology Development Fund Project of Affiliated Hospital of Xuzhou Medical University (Grant Number XYFM202247); Joint Fund of the Huai'an Natural Science Foundation and the Huai'an Commission of Health (Grant Number HABL202242), Natural science research plan of Huai'an (Grant Number HAB202116).

## CONFLICT OF INTEREST STATEMENT

The authors declare no conflict of interest.

## Supporting information


Appendix S1

## Data Availability

The data supporting the findings of this study are available within the article.
