# Supplementary material for: Inhibition of MiR‐106b‐5p mediated by exosomes mitigates acute kidney injury by modulating transmissible endoplasmic reticulum stress and M1 macrophage polarization
Source: J Cell Mol Med. 2023 Jul 20;27(19):2876–89. doi: 10.1111/jcmm.17848 (PMC10538271; doi:10.1111/jcmm.17848)
Supplement: Supplementary file 1 — Appendix S1 [file JCMM-27-2876-s001.docx]

**Inhibition of MiR-106b-5p mediated by exosomes mitigates acute kidney injury by modulating endoplasmic reticulum stress and M1 macrophage polarization**

Xiang Li ^1,2†^, Yanan Zhong^1†^, Rui Yue ^1†^, Juan Xie ^1^, Yiyuan Zhang ^1^, Yongtao Lin ^1, 3^*, Hailun Li^1*^, Yong Xu ^1^*, Donghui Zheng ^1^*

1 Department of Nephrology, The Affiliated Huai'an Hospital of Xuzhou Medical University and Huai'an Second People's Hospital, Huai'an, China

2 Department of Clinical Laboratory, The Affiliated Huai'an Hospital of Xuzhou Medical University and Huai'an Second People's Hospital, Huai'an, China

3 School of Nursing and Midwifery, Jiangsu College of Nursing, Huai’an, China

***Correspondence:**

Hailun Li (lihailun101@163.com)

Yong Xu (haeyxy1@126.com)

Donghui Zheng (haeyzdh@163.com)

†These authors have contributed equally to this research work

**Contents**

**Supplementary Materials and Methods**………………………………………**S3**

**Protein levels of ER stress markers (Figure S1)**……………………………**S6**

**GRP78 and CHOP expression levels (Figure S2)** ………………................**S6**

**Detection of exosomes markers (Figure S3)**……...………………...…...….**S7**

**GRP78 and CHOP levels in THP-1 macrophages (Figure S4)**…………….**S7**

**Expression levels of ATL3, GRP78, and CHOP under miR-106b-5p mimic treatment (Figure S5)**..………...………………………………………………...**S7**

**Expression levels of ATL3, GRP78, and CHOP under miR-106b-5p inhibitor treatment (Figure S6)**……………………………………………...….**S8**

**Levels of ATL3, GRP78, and CHOP in kidney (Figure S7)**..…………....….**S8**

**Analysis of the correlation (Figure S8)** …………...................................….**S8**

**Eight differentially upregulated microRNAs (Figure S9)**. ........................**S9**

**GRP78, CHOP levels after tunicamycin treatment (Figure S10)** .............**S9**

**ATL3 levels in macrophages after exosomes treatment (Figure S11)** ..**S11**

**Primers for quantitative RT-PCR (Table S1)** …………..........................…..**S9**

**Characteristics of CSA-AKI and non-CSA-AKI (Table S2)** .................….**S10**

**Reference**.………………....................................................................….….**S12**

**Supplementary Materials and Methods**

1. **Transfection with miRNA mimics and inhibitors**

MiR-106b-5p mimic or control mimic (RiboBio, China) and miR-106b-5p inhibitor or negative control (NC) inhibitor (RiboBio, China) were transfected into mRTEC. The sequences of the miR-106b-5p mimics were miR-106b-5p (5’-TAAAGTGCTGACAGTGCAGAT-3’ and 3’-ATTTCACGACTGTCACGTCTA-5’). The sequence of the miR-106b-5p inhibitor was 5’-ATCTGCACTGTCAGCACTTTA -3’).

1. **RNA extraction and RT-PCR**

Total RNA was extracted from cells, exosomes, or tissues using TRIzol reagent (15596018, Thermo) according to manufacturer instructions and reverse transcribed into cDNA using EntiLink™ reverse transcriptase or EntiLink™ 1^st^ strand cDNA synthesis kit (ELK Biotechnology, China) and qPCR analyses done on a Quant Studio 6 Flex System (Life technologies) using SYBR green qPCR mix (ELK Biotechnology, China). Fold gene expression changes were determined using the 2-ΔCT method [2] using GAPDH or U6 as reference genes. The primers were listed in Table S1.

1. **Western blot**

Cells, kidney tissues, and exosomes were lysed using RIPA lysis buffer (AS1137, ASPEN) supplemented with a protease inhibitor cocktail (ab141032, Abcam). Total protein concentration was measured using a BCA detection kit (AS1086, ASPEN). Protein samples were resolved on 10% SDS-PAGE and transferred onto PVDF membranes (FFP26, Beyotime). Membranes were then blocked using 5% skimmed milk for 2 h at room temperature and then incubated in anti-GRP78 (SAB5700613, Sigma), anti-CHOP (SAB4500631, Sigma-Aldrich), anti-CD63 (ab216130, Abcam), anti-TSG101 (ab125011, Abcam), anti-Alix (ab275377, Abcam), and anti-ATL3 (ab117819, Abcam) primary antibodies at 4 °C overnight. Finally, signal was developed and imaged on a chemiluminescence detection system (Bio-Rad, USA). GADPH (ab181602, Abcam) was used as loading control.

1. **MiRNA sequencing**

Total exosomal miRNA was extracted using a miRNeasy Micro Kit (QIAGEN, Germany). Libraries were then prepared using a SMARTer smRNA-Seq kit (Clontech, USA) and the miRNA spectrum analyzed on an Illumina Novaeq 6000 (Illumina, USA) platform according to manufacturer guidelines. Data were analyzed by multiple change and *P*-value.

1. **Luciferase reporter assay**

Wild type (WT) and mutant (Mut) ATL3 reporter genes were purchased from ELK biotechnology (Wuhan, China). Plasmids were co-transfected with miR-106b-5p mimics, inhibitors, or corresponding controls into HEK 293T cells (10^6^ cells per well) on a six-well plate using Lipofectamine 2000 (Synthgene, China) according to manufacturer guidelines. After 24 h, luciferase activity normalized to Renilla luminescence was detected using a luciferase assay kit (Biyuntian, China) following manufacturer guidelines.

1. **MiRNA pull-down**

Interaction between miR-106b-5p and its potential target, ATL3, was assessed using miRNA pull-down analysis. A biotin-labeled miR-106b-5p probe (Bio-miR-106b-5p) and a negative control probe (Bio-NC) were purchased from CloudSeq Biotech Inc. (Shanghai, China). Briefly, cells were transfected with the biotin-conjugated probes, lysed with lysis buffer, and then incubated with streptavidin magnetic beads (Invitrogen) at 4 ℃ for 4 h. Bound RNA was then purified using TRIzol and ATL3 mRNA enrichment assessed using RT-PCR.

1. **Immunofluorescence**

Tissue sections were incubated with anti-F4/80 primary antibody (ab6640, Abcam) at 4 ℃ overnight. They were then incubated with the relevant secondary antibody (ab6721, Abcam) for 30 min at room temperature and imaged under an optical microscope.


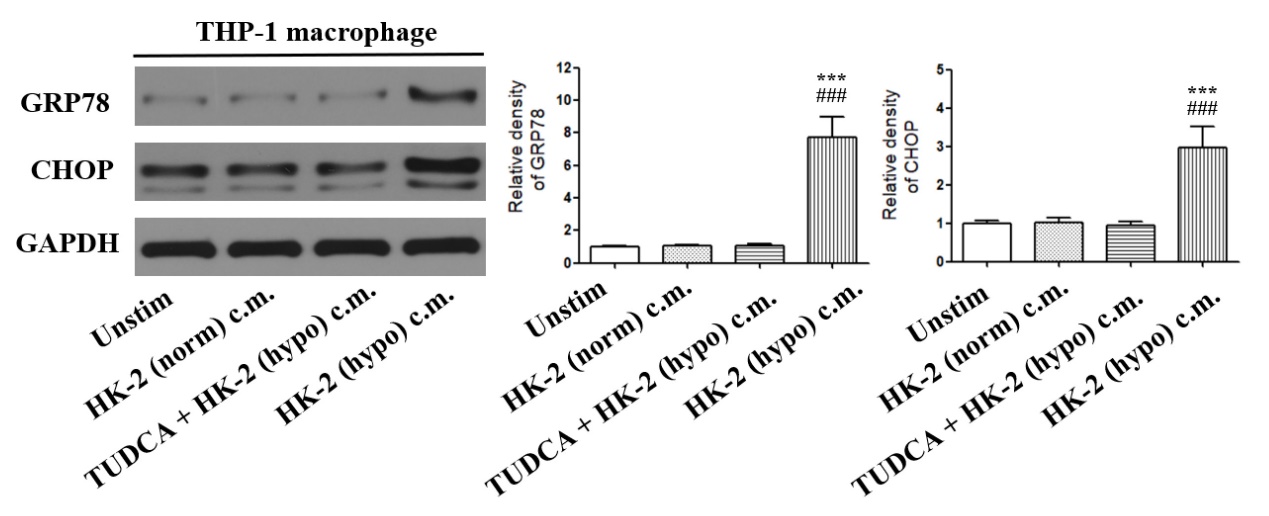


**Figure S1**. Western blot analysis of protein levels of the ER stress markers in THP-1 macrophages under treatment of different conditioned media.


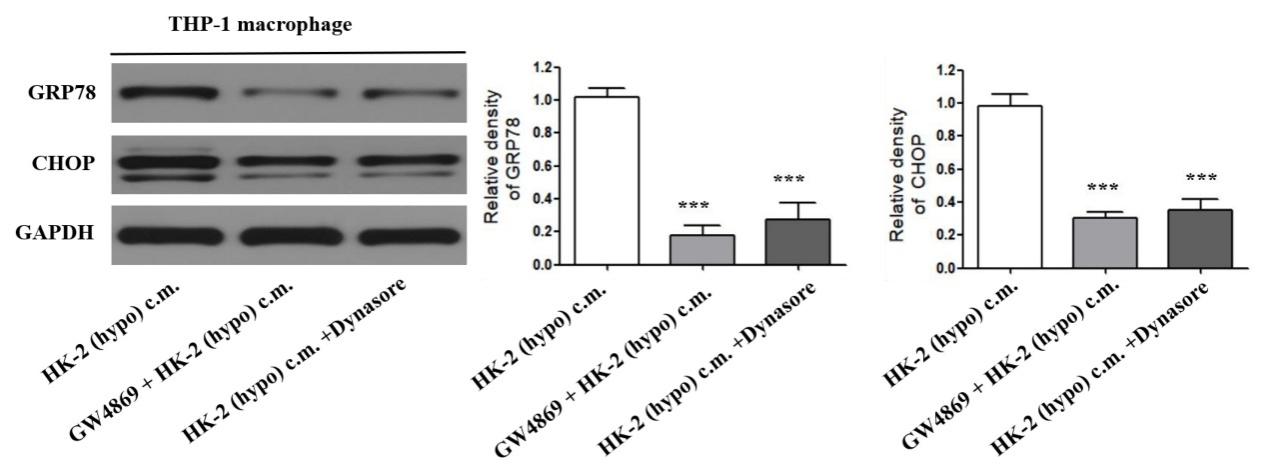


**Figure S2**. Western blot analysis of GRP78 and CHOP expression levels in THP-1 macrophages cultured in conditioned media from HK-2 cells pretreated with GW4869 (10 µM) or Dynasore (50 µM) for 12 h. *** indicates P <0.001 compared with the HK-2 (hypo) c.m. group.


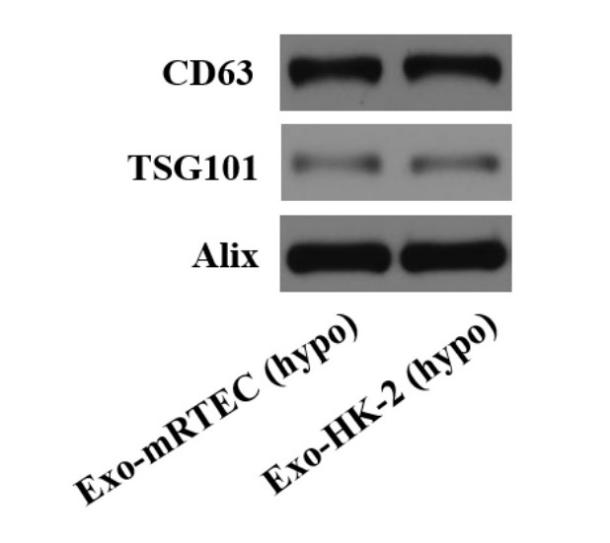


**Figure S3**. Detection of exosomes markers, CD63, TSG101, and Alix examined by western blot.


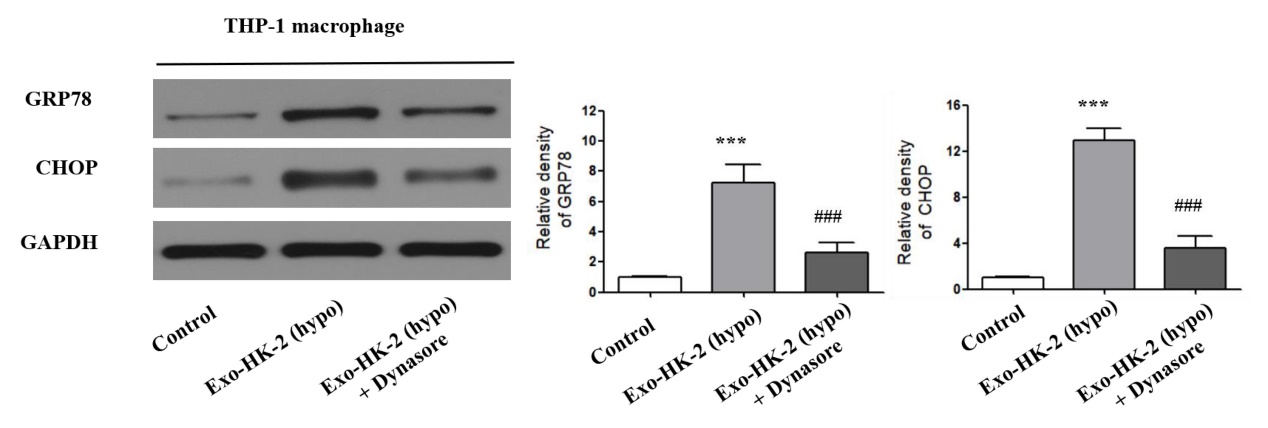


**Figure S4**. Western blot analysis of GRP78 and CHOP levels in recipient THP-1 macrophages incubated with HK-2-derived exosomes (30 ng/mL) for 12 h.


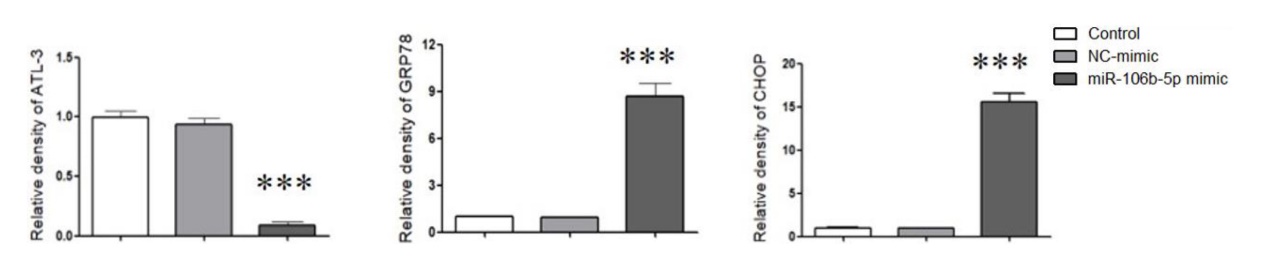


**Figure S5**. Western blot analysis of the expression of ATL3, GRP78, and CHOP in RAW264.7 macrophages incubated with the exosomes from mRTECs transfected with miR-106b-5p mimic or NC-mimic for 12 h. Control exosomes were isolated from mRTECs with plasmid-free transfection.


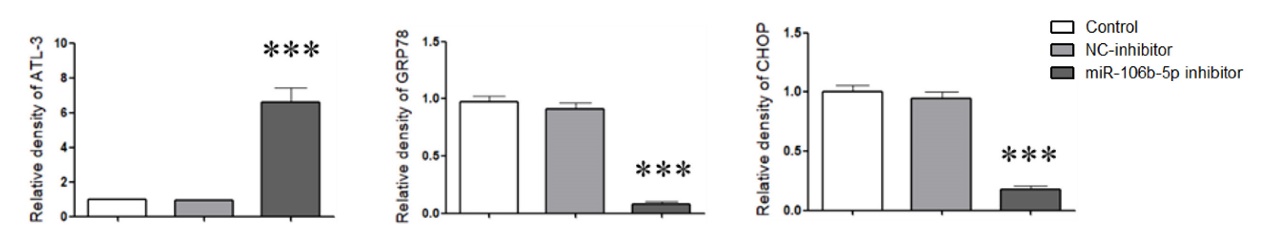


**Figure S6**. Western blot analysis of protein levels of ATL3, GRP78, and CHOP in RAW264.7 macrophages incubated with the exosomes from mRTECs transfected with miR-106b-5p inhibitor or the NC-inhibitor for 12 h, followed by H/R.


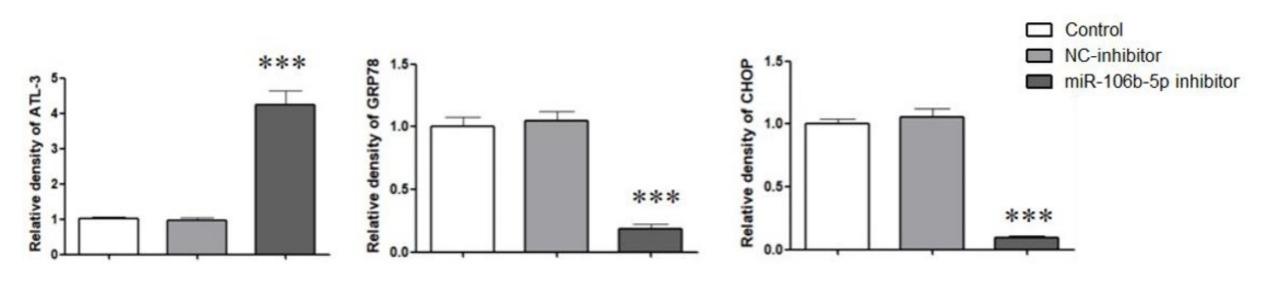


**Figure S7**. Western blot analysis of the expression of ATL3, GRP78, and CHOP in kidney tissues after various treatments.


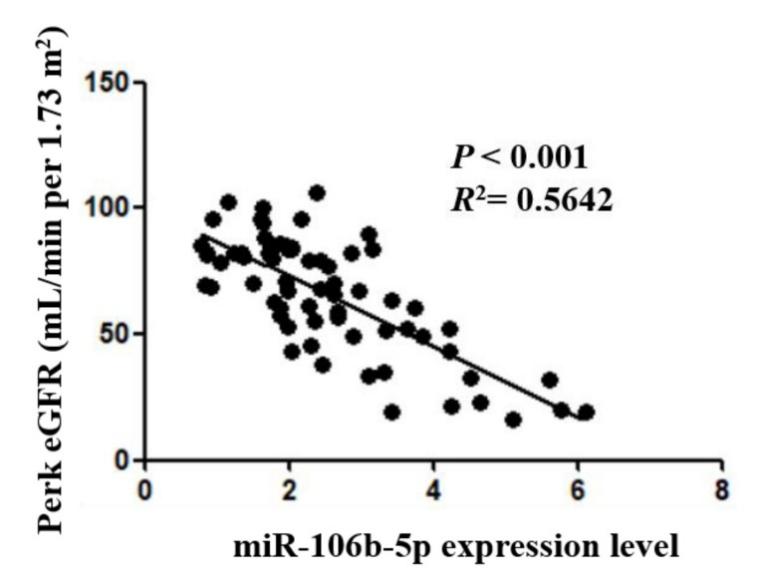


**Figure S8**. Analysis of the correlation between miR-106b-5p levels and peak eGFR levels.


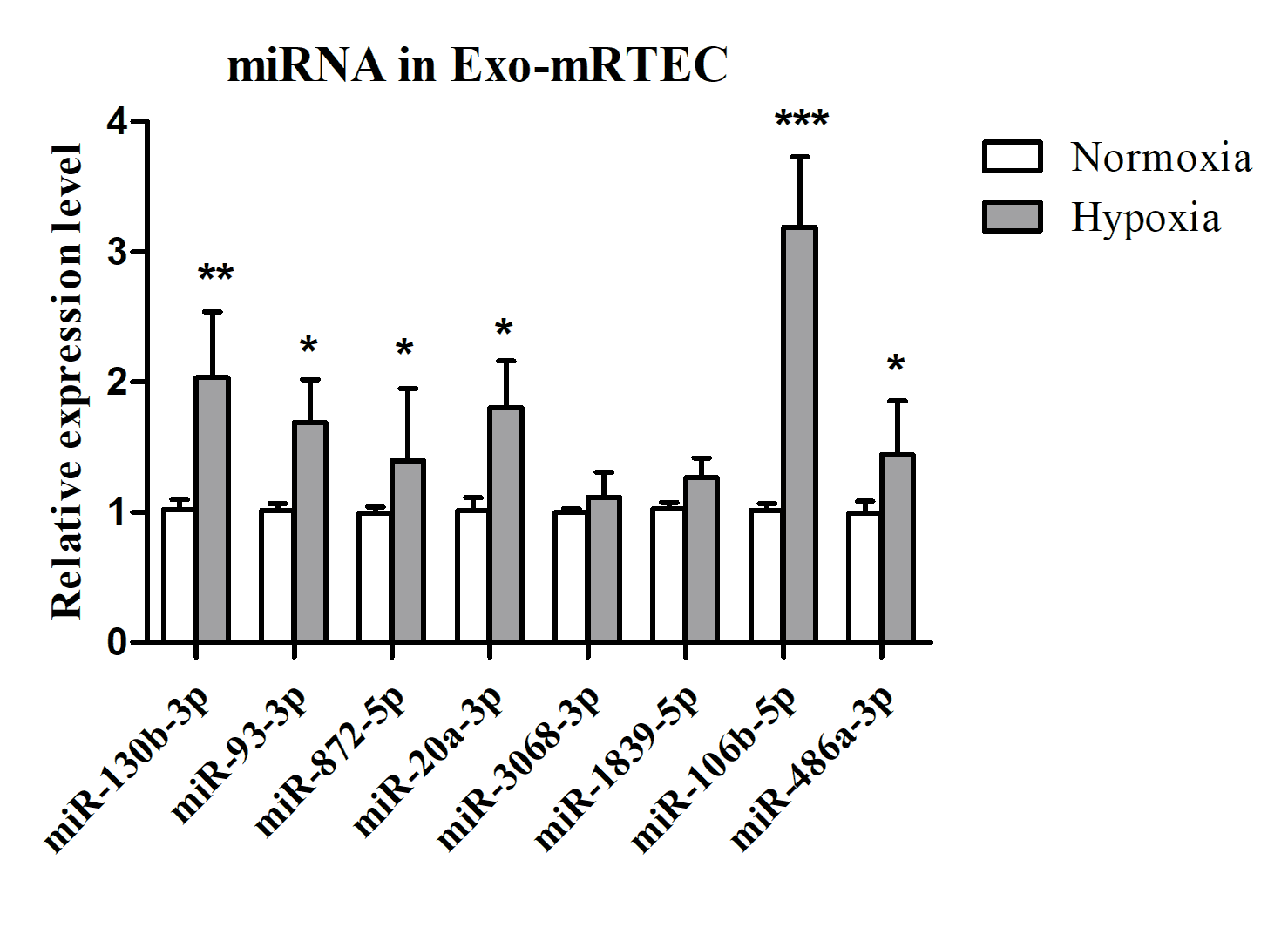


**Figure S9**. The relative expression levels of eight differentially upregulated microRNAs in exosomes derived from mRTECs after hypoxia or normoxia treatment analyzed by qRT-pCR analysis.


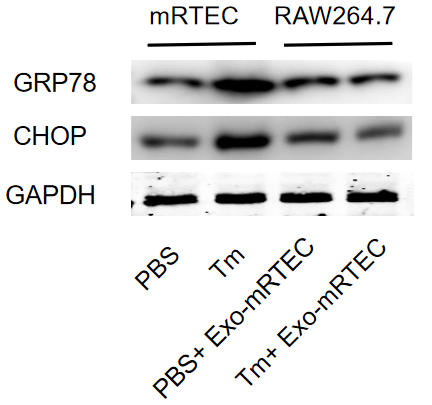


**Figure S10**. Western blot analysis of GRP78 and CHOP expression levels in the mRTEC and RAW264.7 cells after the treatment of PBS, tunicamycin (Tm), mRTECs-derived exosomes plus PBS, and mRTECs-derived exosomes plus Tm.


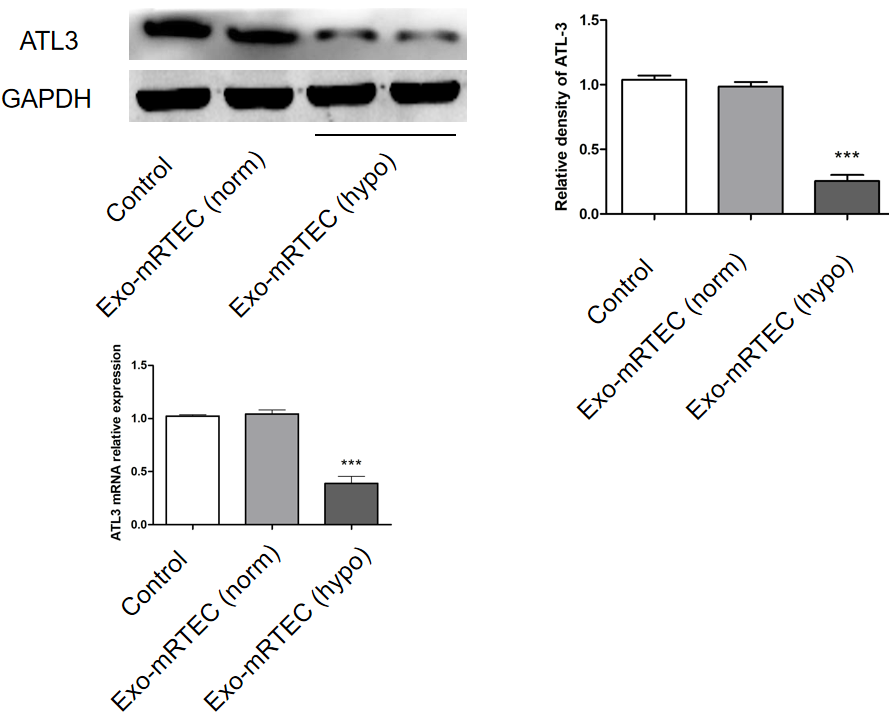


**Figure S11**. Western blot analysis and qRT-PCR detection of ATL3 expression levels in macrophages after the treatment of mRTECs-derived exosomes (norm) and mRTECs-derived exosomes (hypo).

**Table S1 Primers for quantitative RT-PCR**

| **Primer** | **Primersequence,5′–3′** | | |
| --- | --- | --- | --- |
|  |  | **Sense** | **Antisense** |
| Mouse-TNF-α |  | GTACCTTGTCTACTCCCAGGTTCTC | GTCTAAGTACTTGGGCAGATTGACC |
| Mouse-IL-1β |  | GGGCCTCAAAGGAAAGAATCT | GAGGTGCTGATGTACCAGTTGG |
| Mouse-iNOS |  | ACATCAGGTCGGCCATCACT | CAGAGGCAGCACATCAAAGC |
| Mouse-Ccl2 |  | CACTCACCTGCTGCTACTCATTC | CCTTCTTGGGGTCAGCACAG |
| Mouse-ATL3 |  | GAACAACATTCCTTTGAGCTAGAAG | CCACCTTCCTTCTGAGAATATAAGT |
| Mouse-GAPDH |  | TGAAGGGTGGAGCCAAAAG | AGTCTTCTGGGTGGCAGTGAT |
| miR-106b-5p |  | TGGAGTGCTGACAGTGCAGAT | CTCAACTGGTGTCGTGGAGTC |
| U6 |  | CTCGCTTCGGCAGCACAT | AACGCTTCACGAATTTGCGT |
| H-INOS |  | GCAGGACTCACAGCCTTTGG | GGCTGGATGTCGGACTTTGT |
| H-CCL2 |  | CTCATAGCAGCCACCTTCATTC | GATCACAGCTTCTTTGGGACAC |
| H-IL-1β |  | ACGATGCACCTGTACGATCACT | GAGAACACCACTTGTTGCTCCA |
| H-TNF-a |  | CTGTAGCCCATGTTGTAGCAAAC | TGAAGAGGACCTGGGAGTAGATG |
| H-GAPDH |  | CATCATCCCTGCCTCTACTGG | GTGGGTGTCGCTGTTGAAGTC |

| **Table S2 Clinical characteristics of CSA-AKI and non-CSA-AKI patients** | | | |
| --- | --- | --- | --- |
|  | non CSA-AKI  (n = 30) | CSA-AKI  (n = 36) | *P* value |
| Sex, male, n (%) | 19 (63.3) | 21 (58.3) | 0.212 |
| Age (years) | 49.31 ± 6.54 | 51.80 ± 7.67 | 0.346 |
| BMI (kg/m^2^) | 23.67 + 3.22 | 24.02 + 3.55 | 0.189 |
| Diabetes, n (%) | 11 (36.7) | 14 (38.9) | 0.552 |
| Hypertension, n (%) | 16 (53.3) | 20 (55.6) | 0.106 |
| Current smoking, n (%) | 10 (33.3) | 14 (38.9) | 0.149 |
| CPB time, min, median (IQR) | 115 (83–125) | 120 (86–142) | 0.136 |
| CPB time > 120 min, n (%) | 9 (30.0) | 17 (47.2) | 0.004 |
| Hospitalized time, d, median (IQR) | 18 (12–21) | 14 (13–18) | 0.031 |
| Prior operation |  |  |  |
| Baseline SCr (μmol/L) | 63.44 ± 15.01 | 65.72 ± 15.53 | 0.075 |
| Baseline eGFR  (mL/min per 1.73 m2) | 87.80 + 5.60 | 87.73 + 7.07 | 0.323 |
| Hb (g/L) | 135.6 + 19.1 | 137.2 + 22.7 | 0.486 |
|  |  |  |  |
| Post operation |  |  |  |
| Perk SCr (μmol/L) | 66.91 ± 13.08 | 174.56 ± 89.31 | < 0.001 |
| Stage I |  | 108.81 ± 21.71 |  |
| Stage II | - | 157.24 ± 40.58 |  |
| Stage III | - | 312.62 ± 38.11 |  |
| Perk (eGFRmL/min per 1.73 m^2^) | 85.70 + 6.21 | 48.438 + 16.87 | < 0.001 |
| Stage I | - | 62.49 ± 5.49 |  |
| Stage II | - | 47.80 ± 8.81 |  |
| Stage III | - | 24.41 ± 6.86 |  |
| Hb (g/L) | 94.3 + 13.8 | 91.3 + 16.9 | 0.113 |
| miR-106b-5p (2^−∆∆ct^) | 1.77 ± 0.62 | 3.23 ± 1.06 | < 0.001 |

BMI: body mass index, CPB: cardiopulmonary bypass, SCr: serum creatinine, eGFR: estimated glomerular filtration rate. Preoperative SCr was measured within two weeks before surgery and when measured multiple times, the highest value was used as baseline. Postoperative SCr was measured within 48 h after surgery and if measured multiple times, the highest value was used as peak value. Statistical differences were compared using unpaired Student’s t-test (*P* value).

**References**

[1] Y. Liang, L. Liang, Z. Liu, et al., Inhibition of IRE1/JNK pathway in HK-2 cells subjected to hypoxia-reoxygenation attenuates mesangial cells-derived extracellular matrix production, J Cell Mol Med. 24 (22) (2020) 13408-13420.

[2] N. Zhou, Z. Xu, X. Li, et al., Schwann Cell-Derived Exosomes Induce the Differentiation of Human Adipose-Derived Stem Cells into Schwann Cells, Front Mol Biosci. 8 (1) (2021) e835135.
